# The Effect of Electrical Stimulation Strength Training on Lower Limb Muscle Activation Characteristics During the Jump Smash Performance in Badminton Based on the EMS and EMG Sensors

**DOI:** 10.3390/s25020577

**Published:** 2025-01-20

**Authors:** Xinyu Lin, Yimin Hu, Yi Sheng

**Affiliations:** 1 School of Exercise and Health, University of Sport, Shanghai 200438, China; 2421517010@sus.edu.cn; 2 School of Athletic Performance, University of Sport, Shanghai 200438, China; 2321151040@sus.edu.cn

**Keywords:** electrical muscle stimulation, strength training, lower limb muscle, activation characteristics, jump smash performance, electromyography, badminton

## Abstract

This study investigates the effects of electrical stimulation (EMS) combined with strength training on lower limb muscle activation and badminton jump performance, specifically during the “jump smash” movement. A total of 25 male badminton players, with a minimum of three years of professional training experience and no history of lower limb injuries, participated in the study. Participants underwent three distinct conditions: baseline testing, strength training, and EMS combined with strength training. Each participant performed specific jump tests, including the jump smash and static squat jump, under each condition. Muscle activation was measured using electromyography (EMG) sensors to assess changes in the activation of key lower limb muscles. The EMS intervention involved targeted electrical pulses designed to stimulate both superficial and deep muscle fibers, aiming to enhance explosive strength and coordination in the lower limbs. The results revealed that the EMS + strength condition significantly improved performance in both the jump smash and static squat jump, as compared to the baseline and strength-only conditions (*F* = 3.39, *p* = 0.042; *F* = 3.67, *p* = 0.033, respectively). Additionally, increased activation of the rectus femoris (RF) was observed in the EMS + strength condition, indicating improved muscle recruitment and synchronization, likely due to the activation of fast-twitch fibers. No significant differences were found in the eccentric-concentric squat jump (*F* = 0.59, *p* = 0.561). The findings suggest that EMS, when combined with strength training, is an effective method for enhancing lower limb explosiveness and muscle activation in badminton players, offering a promising training approach for improving performance in high-intensity, explosive movements.

## 1. Introduction

Electrical stimulation training has gradually become an important training tool in both competitive sports and rehabilitation. By applying electrical pulses to the muscles, it promotes muscle fiber contraction, thereby enhancing muscle strength and endurance [1,2]. Initially used in rehabilitative medicine to maintain or develop muscle mass, especially in cases of central nervous system impairment, electrical stimulation has recently gained widespread attention in the field of sports science. Notably, in strength-based sports such as rugby and sprinting, it has yielded significant results [3,4]. In these sports, electrical stimulation has been shown to effectively increase athletes’ muscle strength, thereby improving their performance [5].

Badminton, as a sport that combines both technical skill and explosive power, places high demands on the strength and coordination of the lower limbs. This is particularly evident in the “jump smash”, a key movement where athletes must explosively propel themselves into the air and coordinate their upper limbs for a powerful strike [6,7]. The introduction of electrical stimulation training aims to further enhance athletes’ lower limb explosiveness and endurance, thereby improving their jumping ability and stability in competition [8,9]. Jump kill is one of the most threatening offensive movements in badminton, which has extremely important strategic significance. As a highly explosive technique, it can quickly change the rhythm of the game and bring great pressure to the opponent [10]. Through jumping, athletes are able to hit the badminton ball at high altitude and combine the ball speed and angle through powerful swinging action to create an attack that is difficult to defend. Therefore, jumping kills are not only able to score directly, but also create opportunities for the next attack by constantly applying pressure and making the opponent’s defense vulnerable. In addition, the jump kill is a technique that can effectively use explosive power, especially when facing opponents with quick reactions and high defensive intensity. It can break through the opponent’s defensive strategy and disrupt their rhythm. The jump kill not only demonstrates a player’s physical ability and skill, but also reflects the player’s on-court intelligence, as it requires precise strokes and quick decision-making to be completed in the air, making it one of the indispensable core techniques in badminton [11].

However, traditional resistance training has limitations in increasing lower limb strength, such as a lack of variety in exercises and an inability to fully activate the muscles’ maximal potential, especially the deep muscle groups [12]. Electrical stimulation can directly activate muscle fibers, particularly fast-twitch fibers, through electrical pulses, enabling recruitment of these fibers even under low intensity, thus improving lower limb strength performance [13].

Electrical stimulation training is able to activate deep muscle groups and rapidly contract muscle fibers through precise electrical pulse frequency and current intensity, which is difficult to do in traditional training methods. Studies have shown that electrical stimulation can not only enhance muscle endurance, but also effectively promote the improvement of muscle strength and explosive force, especially for the athletes’ jumping ability, to improve the obvious effect [14]. In badminton, athletes’ jumping height and explosive power directly affect their attacking efficiency and reaction speed in the game; therefore, electrical stimulation training helps athletes to improve their performance in the game. In addition, the advantage of electrical stimulation training is that it can be used as an auxiliary training tool, which can be combined with traditional training methods to achieve better results. Studies have shown that electrical stimulation can be performed after rest or training of athletes to achieve the effect of enhancing muscle endurance and strength by strengthening muscle recovery and improving muscle depth activation [15]. This type of training is not only applicable to high-level athletes in competitive sports, but can also provide an effective means of muscle rehabilitation for injured or post-operative patients during the rehabilitation process. Further studies have shown that electrical stimulation training can improve muscle fatigue recovery in athletes, increase muscle metabolism, and reduce lactic acid accumulation during exercise, thus delaying the onset of fatigue [16]. This is particularly important for sports requiring high-frequency explosive power such as badminton. In badminton, athletes need to perform multiple explosive movements in a short period of time, and electrical stimulation training can enhance muscular endurance and slow down the accumulation of fatigue, thus improving athletes’ performance.

Existing research indicates that electrical stimulation training can effectively improve lower limb muscle strength and endurance. A study by Draghici [17] and colleagues demonstrated that electrical stimulation intervention in rowers not only significantly enhanced their leg strength and endurance but also improved fast-twitch fiber activation and muscular endurance through dynamic and static electrical stimulation methods [18]. This approach addresses the limitation of traditional resistance training, where neural impulse regulation is restricted, preventing full muscle potential activation. As electrical stimulation technology continues to advance, combining it with traditional resistance training has become increasingly mainstream, especially in strength- and endurance-dominant sports such as rowing [19]. By incorporating electrical stimulation, both superficial and deep muscles can be targeted, allowing athletes to achieve comprehensive strength development, thereby enhancing their specific performance abilities [20].

The electromyography sensor (EMG) is an advanced tool widely used in sports science and biomechanics research to monitor and analyze the electrical activity of muscles in real time. By placing electrodes on the surface of the skin, EMG is able to accurately capture the electrical signals generated by muscle fibers during contraction and relaxation. These signals reflect the degree of muscle activation, the type of contraction, and the coordination of different muscle groups during exercise, and thus have important applications in sports training and assessment [21]. In competitive sports, EMG is often used to assess the muscle activation characteristics of athletes under different training and exercise modes, especially for lower limb muscle groups. For sports such as badminton, which require fast and explosive power, EMG can help coaches and athletes to understand the working status of lower limb muscles during key movements, such as the activation of quadriceps, gluteal muscles, and calf muscle groups during jumping and hitting. These data can provide a scientific basis for the development of training programs and optimize athletes’ technical movements. In addition, EMG is widely used in rehabilitation medicine to assess and restore muscle function. During the recovery process after injury or surgery, by analyzing EMG signals, rehabilitation experts are able to accurately determine the recovery process of damaged muscles and formulate personalized rehabilitation plans [22]. EMG technology is also used to monitor the occurrence of muscle fatigue, and by analyzing the trend in EMG changes, training intensity can be adjusted to avoid muscle damage or fatigue due to over-training in athletes [23].

However, research on electrical stimulation training for badminton players, particularly for complex movements like the jump smash, remains limited. This movement involves both lower limb explosive strength and overall body coordination. Therefore, this study aims to explore the effects of electrical stimulation training on the lower limb muscle activation characteristics of badminton players during the “jump smash”. Through comparisons of baseline testing, strength training, and combined electrical stimulation and strength training, this study seeks to provide an effective lower limb strength training method for badminton athletes and offer theoretical support for the application of electrical stimulation in competitive sports.

## 2. Participants and Method

### 2.1. Participants

This study recruited 25 male badminton players with a minimum of three years of professional training experience and no history of lower limb injuries (Table 1). All participants provided informed consent prior to the study, which was approved by the institutional ethics committee of Shanghai University of Sport and conducted in compliance with the Declaration of Helsinki. Each participant underwent testing under all training conditions, allowing evaluation of muscle activation characteristics across different conditions within the same cohort.

#### 2.1.1. Inclusion Criteria

Male, aged 18–30 years. At least three years of professional badminton training experience. No history of lower limb injuries or other conditions affecting normal motor function. Ability to participate in the study sessions punctually and adhere to the training protocol.

#### 2.1.2. Exclusion Criteria

Cardiovascular or neuromuscular diseases that could compromise experimental safety. History of severe lower limb injury within the past six months. Allergic reactions or intolerance to electrical stimulation. History of epilepsy or related disorders. Inability to participate punctually in training sessions or usage of medication affecting muscle function during the study period.

### 2.2. Experimental Design

(1) Baseline Testing (Control Condition).

Objective: To assess the participant’s initial muscle activation characteristics and jump performance without any training intervention.

Procedure: Participants performed baseline testing, which included both static squat jumps and eccentric-concentric squat jumps. The jump height and muscle activation characteristics during these tests were recorded as baseline data.

Muscle activation metrics: The muscle activation was recorded using EMG sensors, and key metrics such as mean EMG amplitude, root mean square (RMS) amplitude, and integrated EMG (iEMG) were calculated and normalized to the maximum values recorded.

Outcome measures: Jump height and muscle activation patterns of the quadriceps, hamstrings, and calf muscles during both the squat jump and the jump smash were measured.

(2) Strength Training Only (Strength Only Condition).

Objective: To assess the effects of traditional strength training on lower limb muscle activation characteristics and jump performance.

Procedure: Participants performed a resistance training protocol consisting of barbell squats at 65% of their one-repetition maximum (1RM) for four sets of 20 repetitions, with a 30 s rest interval between sets. Immediately following the strength training, participants performed the jump smash and squat tests again to measure jump height and muscle activation characteristics.

Outcome Measures: Muscle activation metrics and jump height were assessed as per the baseline testing. This condition served as the control for comparing the effects of EMS combined with strength training.

(3) Combined Electrical Muscle Stimulation (EMS) and Strength Training (EMS + Strength Condition).

Objective: To evaluate the effects of combining EMS with traditional strength training on lower limb muscle activation and jump performance.

Procedure: In this condition, EMS was applied during the weighted squats. The EMS protocol delivered fixed-intensity electrical pulses during the eccentric, stretch-reflex, and concentric phases of the squat. During the standing (relaxed) phase, the EMS switched to a low-intensity relaxation mode. This combination has been hypothesized to enhance muscle activation and facilitate greater performance improvements. After completing the EMS-enhanced strength training, participants underwent the same jump smash and squat tests to assess muscle activation and performance.

Outcome Measures: The same muscle activation metrics and jump performance measures were recorded and compared with the baseline and strength-only conditions.

(4) Electrode Placement.

For the EMG sensors, electrodes were placed on the skin over the primary muscles involved in the lower limb movements during the squat and jump smash. The placement followed the standard guidelines recommended for accurate and reliable muscle activation measurements in strength training and jump performance. Specifically, the electrodes were placed as follows:

Quadriceps (rectus femoris): Electrodes were positioned on the anterior thigh, at the midpoint between the iliac crest and the patella.

Hamstrings (biceps femoris): Electrodes were placed on the posterior thigh, at the midpoint between the ischial tuberosity and the lateral condyle of the femur.

Calf muscles (gastrocnemius): Electrodes were placed on the mid-belly of the calf muscles, over the gastrocnemius, avoiding the Achilles tendon area.

Gluteus maximus: Electrodes were positioned on the upper part of the gluteal region, in line with the iliac crest.

The distance between electrodes on each muscle group was typically 2 to 4 cm to ensure optimal signal detection while avoiding cross-talk from neighboring muscles. In cases of muscle overlap or complex joint movements, electrodes were placed to maximize the accuracy of muscle-specific activation patterns.

### 2.3. Experimental Procedure

Each experimental session began with a 10 min warm-up, consisting of five minutes of jogging and five minutes of dynamic stretching of the lower limbs, ensuring sufficient muscle and ligament preparation. Participants then completed tests under the three training conditions sequentially, with lower limb muscle activation characteristics recorded using Noraxon surface electromyography (EMG) equipment.

### 2.4. Electrical Stimulation Parameters

**Figure 1:**
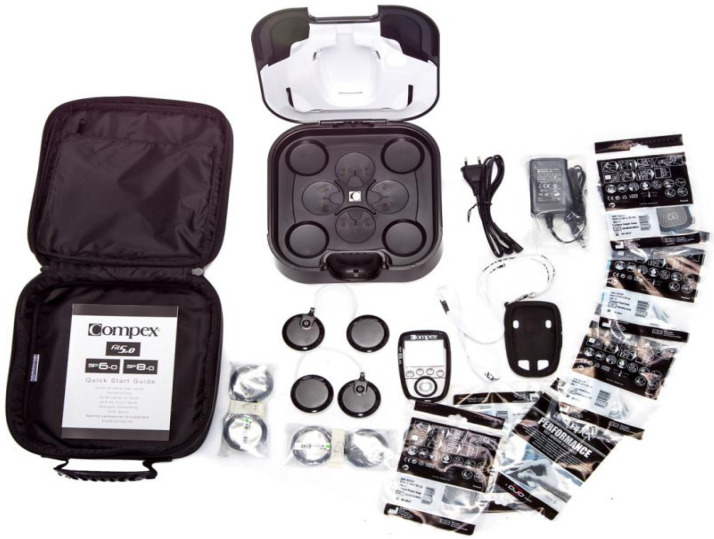
Compex SP 8.0 Electrical Stimulation System.

In the EMS + strength condition, EMS was applied to the quadriceps using a Compex SP 8.0 electrical stimulation device (Figure 1). Specific EMS parameters were as follows [24,25]:

(1) Current type: Biphasic symmetric square wave.
(2) Frequency: 100 Hz, selected to activate fast-twitch muscle fibers and optimize strength training outcomes.
(3) Pulse duration: 300 µs, ensuring effective deep muscle stimulation.
(4) Intensity: Calibrated individually based on initial pain threshold testing, typically set between 25 and 35 mA to achieve at least 60% of maximum voluntary contraction (MVC).
(5) Electrode placement: Self-adhesive electrodes were placed on the quadriceps muscles of both knees, with each electrode measuring 25 cm^2^ (5 cm × 5 cm) or 50 cm^2^ (10 cm × 5 cm).
(6) Stimulation mode: EMS was configured to an endurance mode, delivering fixed-intensity pulses during the eccentric, stretch-reflex, and concentric phases of each squat cycle, with a transition to low-intensity relaxation mode (10 mA, frequency 3 Hz) upon completion of each cycle.

### 2.5. Measurement Variables

#### 2.5.1. Jump Smash Performance

Jump height was measured using a Qualisys motion capture system (Qualisys AB, Gothenburg, Sweden), which consists of eight infrared cameras (250 Hz) to capture three-dimensional kinematic data during the jump. A total of 39 reflective markers were placed on anatomical landmarks across the body, including the thorax, pelvis, femur, tibia, and foot, to establish joint centers and segment orientations. A three-dimensional kinematic analysis of the lower limb and trunk joints was performed. Jump height was calculated from the vertical velocity of the center of mass (CoM) at takeoff, identified as the moment when the direction of the toe markers shifted abruptly from horizontal to vertical. The position of the overall CoM was derived from the distribution of the CoM of individual body segments, allowing for precise measurement of jump height [26].

#### 2.5.2. Static Squat Jump and Eccentric-Concentric Squat Jump Performance

Jump heights for the static squat jump and eccentric-concentric squat jump were recorded using the Perform Better Jump Testing System (Model: Standard). This compact, precision instrument quickly measures lower limb power and agility. The handheld device displays jump height, air time for single jumps, and provides averages for contact time, height, and air time over four consecutive jumps.

#### 2.5.3. Electromyography (EMG) Data Collection

Muscle activation was recorded using Noraxon surface EMG equipment, capturing signals from four muscles on the right side: vastus medialis (VM), vastus lateralis (VL), rectus femoris (RF), and biceps femoris (BF). Prior to testing, hair was removed, and the skin was cleaned to ensure optimal electrode conductivity. Electrodes were secured with adhesive tape to maintain stable positioning. EMG data were collected for both static squat jumps and eccentric-concentric squat jumps, capturing mean EMG amplitude, root mean square (RMS) amplitude, and integrated EMG (iEMG).

Each EMG metric (mean amplitude, RMS amplitude, and iEMG) was normalized to its respective maximum value for each participant to standardize data across testing sessions.

**Figure 2:**
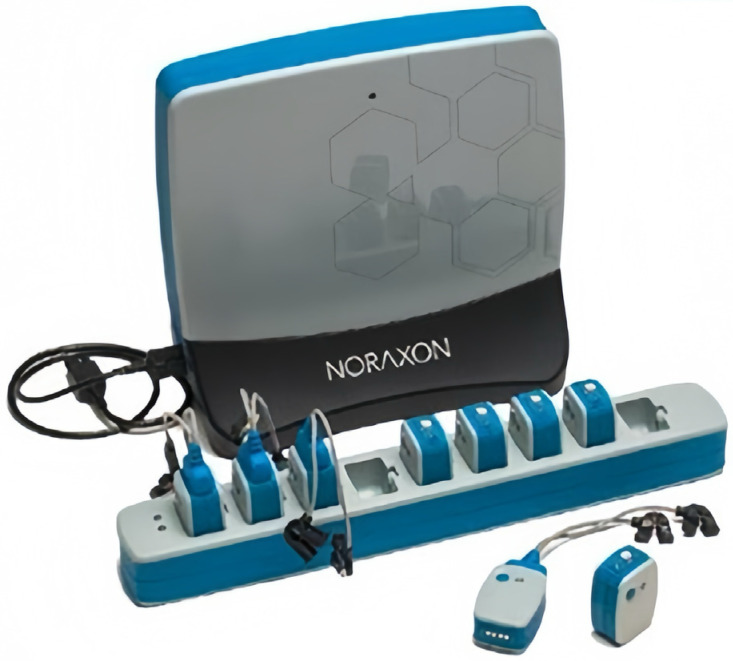
Surface EMG testing and analysis system.

**Figure 3:**
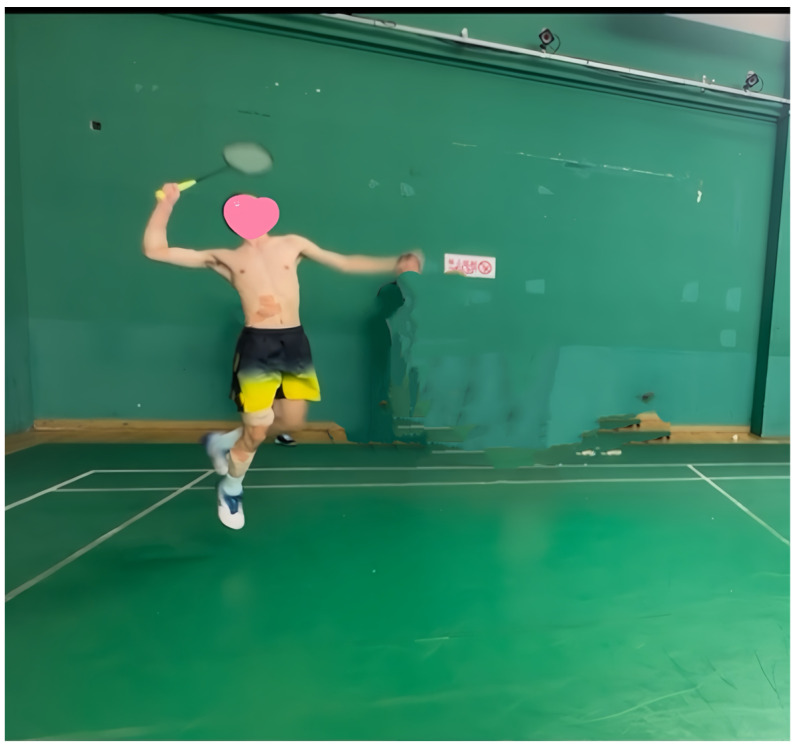
Surface EMG positions and experimental maps of badminton’s “jump smash” maneuvers.

Surface EMG position and movement field maps are shown in Figure 2 and Figure 3.

### 2.6. Statistical Analysis

Data were analyzed using SPSS 26.0 software (SPSS Inc., Chicago, IL, USA). Outliers were first identified using box plots to ensure data accuracy and prevent distortions in statistical outcomes. The normality of the data distribution was evaluated using the Shapiro–Wilk test, which was applied to each variable to assess whether the data followed a normal distribution. To compare the differences in muscle activation and jump performance across the three training conditions (baseline, strength training, and EMS + strength training), a one-way repeated measures analysis of variance (ANOVA) was conducted. This method allowed for the evaluation of within-subject changes over time while controlling for individual variability. Pairwise comparisons were performed following significant ANOVA results, with a Bonferroni correction applied to adjust for multiple comparisons, minimizing the risk of Type I errors. In cases where the assumption of sphericity (equal variances of the differences) was violated, the Greenhouse–Geisser correction was used to adjust the degrees of freedom, providing more accurate results. A significance level of *p* < 0.05 was set for all statistical tests, indicating that results with *p*-values below this threshold were considered statistically significant [27].

## 3. Results

**Figure 4:**
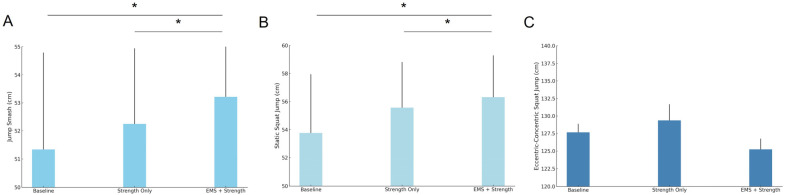
Differences in badminton jump performance under different conditions. (**A**) Jump smash; (**B**) static squat jump; (**C**) eccentric-concentric squat jump; * represents a significant difference.

The differences in badminton jump performance under different conditions showed significant effects for the jump smash and static squat jump (*F* = 3.39, *p* = 0.042; *F* = 3.67, *p* = 0.033, respectively). The EMS + strength condition demonstrated significant improvements compared to both the baseline and strength only conditions. Additionally, the EMS + strength condition also showed significant improvements over strength only for the jump smash and static squat jump. No significant differences were observed for the eccentric-concentric squat jump (*F* = 0.59, *p* = 0.561) (Table 2 and Figure 4).

Regarding the EMG mean values during the static squat jump, a significant effect was found for RF (*F* = 3.44, *p* = 0.040), with higher activation in the EMS + strength condition compared to both the baseline and strength only conditions. No significant differences were found for VL, VM, or BF (Table 3).

For RMS amplitude during the static squat jump, significant differences were observed in RF (*F* = 3.66, *p* = 0.033), with higher activation in the EMS + strength condition compared to both the baseline and strength only conditions. No significant differences were found for VL, VM, or BF (Table 4).

In terms of integrated EMG during the static squat jump, no significant differences were found for VL, RF, VM, or BF across the conditions (Table 5).

For the eccentric-concentric squat jump, no significant differences were found in EMG mean values (all *p* > 0.05), RMS amplitude (all *p* > 0.05), or integrated EMG (all *p* > 0.05) for any muscle group (Table 6, Table 7 and Table 8).

## 4. Discussion

The results of this study indicate that electrical stimulation (EMS) combined with strength training has a significant impact on badminton jump performance and lower limb muscle activation, particularly during the jump smash and static squat jump. The EMS + strength condition showed substantial improvements in jump height compared to both the baseline and strength only conditions. These findings suggest that EMS, when added to traditional resistance training, can enhance muscle performance in explosive movements. However, no significant effects were observed in the eccentric-concentric squat jump, which may be attributed to the specific demands of this exercise or its different muscle activation patterns, as discussed below.

### 4.1. Effect on Jump Performance

The improvement in jump smash and static squat jump performance under the EMS + strength condition is consistent with previous research demonstrating the benefits of electrical stimulation for enhancing muscle strength and explosiveness. The jump smash involves a high degree of lower limb explosiveness and coordination with upper body actions, making it a demanding movement for athletes [28]. Similarly, the static squat jump requires substantial force production, particularly in the lower limbs. The significant improvements observed in both of these exercises in the EMS + strength condition can be attributed to the enhanced recruitment of fast-twitch muscle fibers facilitated by the electrical pulses. These fibers are crucial for explosive movements, and their activation likely contributed to the increased jump height observed in the current study [29]. This aligns with earlier findings that EMS improves explosive performance by increasing fast-twitch fiber recruitment and muscle contraction efficiency. Electrical stimulation (EMS) has been shown in previous studies to be effective in promoting the recruitment of fast muscle fibers and increasing the efficiency of muscle contraction, leading to improved performance, especially in sports requiring explosive power [30]. However, there are studies that have reported the positive effects of electrical stimulation in improving explosive power, but the variability in the findings should not be ignored. Some studies have found a more limited effect of electrical stimulation, possibly due to factors such as differences in stimulation parameters (e.g., frequency and intensity) or insufficient training duration. In contrast, in the present study, the combination of EMS and strength training, by optimizing the frequency and intensity of electrical stimulation as well as the training period, may have achieved a more optimal recruitment of fast-twitch fibers and muscle adaptation, and therefore significantly increased jump height. This phenomenal change may stem from the synergistic effect of electrical stimulation and strength training, which further enhances muscle explosiveness and efficiency through a more precise combination of stimulation and training intensity. This difference reflects the different effects of different experimental designs and individual training backgrounds on the effects of electrical stimulation.

The lack of significant improvements in the eccentric-concentric Squat Jump could be due to the nature of this exercise, which combines both eccentric (muscle lengthening) and concentric (muscle shortening) contractions. The jump smash and static squat jump primarily focus on concentric muscle actions that are more directly influenced by explosive strength training and EMS [31]. On the other hand, the eccentric-concentric squat jump involves more complex muscle coordination and may require a different type of stimulus to elicit improvements. Therefore, it is possible that EMS alone was insufficient to enhance performance in this type of movement, which may require a combination of other training modalities or specific techniques for improving eccentric strength [32]. Previous studies have shown that electrical stimulation (EMS) is effective in enhancing explosive power in concentrated strength training, especially in single centripetal movements such as jumping and rapid pushing. However, the effect is not necessarily so significant for complex movements involving a combination of centrifugal (muscle elongation) and centripetal (muscle contraction) muscle phases [33]. Research has pointed out that the centrifugal contraction phase has unique requirements for muscle strength gains, particularly in terms of muscle control and coordination, and requires special stimulation and training modalities. For example, it has been shown that centrifugal training is effective in increasing muscular strength and endurance, but relying on EMS alone may not be sufficient to significantly improve the performance of this type of compound movement [34]. This explains why centrifugal-centripetal jumps failed to yield significant improvements in the present study. It may be because the movement requires a higher degree of muscle coordination as well as enhancement of centrifugal force, whereas electrical stimulation mainly optimizes centripetal force, and therefore the effects of electrical stimulation may not have been fully exploited in this particular training context. Consistent with other studies, this phenomenon may indicate that EMS stimulation alone is not sufficient to enhance the performance of the centrifugal-centripetal compound movement, and that more specialized training may be required, particularly in terms of centrifugal force enhancement.

### 4.2. EMG and Muscle Activation

Muscle activation patterns during the static squat jump revealed significant differences in the rectus femoris (RF) muscle. Both EMG mean values and RMS amplitude for the RF were higher in the EMS + strength condition compared to the baseline and strength only conditions [35]. The increased muscle activation in the RF suggests that electrical stimulation may have enhanced the coordination and synchronization of muscle fiber discharge. This could be due to the direct stimulation of motor units, particularly the fast-twitch fibers, which are responsible for producing rapid, forceful contractions [36].

The increased activation of RF is also supported by the RMS amplitude data, which indicated a higher level of muscle activity under EMS conditions. RMS amplitude is directly related to the overall intensity of muscle activation, and the increase in this value suggests that the muscle fibers were recruited more effectively during the static squat jump [37]. This result aligns with the hypothesis that EMS improves muscle recruitment and activation by synchronizing the discharge of motor units, which in turn may enhance performance during explosive movements such as jumping.

The effects of electrical stimulation (EMS) on muscle activation typically enhance muscle recruitment and activation by augmenting the synchronized discharge of motor units. For example, EMS was found to be effective in increasing the activation of fast muscle fibers, which are essential for fast and powerful contractions, especially during explosive strength training [38]. Specifically, electrical stimulation enhances muscle coordination and synchronization through direct stimulation of motor units, thereby increasing muscle strength and explosiveness. Consistent with this study, significant activation enhancement of the rectus femoris (RF) muscle in static deep squat jumps reflects the role of electrical stimulation in improving muscle activation efficiency and recruitment. This phenomenon is consistent with previous studies, but there are differences. It has been noted that while electrical stimulation enhances explosive performance in centripetal movements [39], it is less effective than expected in non-centripetal movements, which may be related to the complexity of the movement itself and the requirement for muscle coordination. Unlike these studies, the results of the present study suggest that EMS combined with strength training may effectively enhance static deep squat jump performance by increasing the strength and coordination of muscle activation, especially the recruitment of fast muscle fibers. Thus, the present study further validates the potential of EMS in explosive training, especially in enhancing fast-contracting muscle groups.

In contrast, no significant changes were observed in the vastus lateralis (VL), vastus medialis (VM), or biceps femoris (BF) muscles. These results suggest that the EMS training may have preferentially targeted the RF, likely due to its role in the concentric phase of the squat jump. While the RF is primarily responsible for knee extension during jumping, the VL, VM, and BF muscles play a more supportive role in lower limb stability and balance. It is possible that EMS was less effective at activating these muscles, particularly in comparison to the RF, which is more directly involved in the explosive actions required for the jump [40]. There are differences in the activation effects of electrical stimulation (EMS) on different muscle groups, usually based on the role of the muscle in the movement. The rectus femoris (RF) has a more prominent role in explosive jumping man oeuvres, especially during knee extension, where it is directly involved in the explosive force of the jump and is therefore more susceptible to significant effects of electrical stimulation. In contrast, the lateral femoral (VL), medial femoral (VM) and biceps femoris (BF) muscles mainly play a stabilizing and assisting role, especially in maintaining lower limb balance and controlling posture, and their activation needs are lower. Therefore, EMS may not perform as effectively on these assistive muscles as it does on the rectus femoris. It has also been pointed out that electrical stimulation is more inclined to stimulate those muscles that play a dominant role in explosive movements during training, while the activation effect on supporting muscles is relatively weak [41]. This explains why there was no significant change in the activation of the lateral femoral, medial femoral, and biceps femoris muscles in the present study, reflecting the fact that the effect of electrical stimulation training was more focused on enhancing the activation of the dominant muscles than on the performance of the supporting muscle groups. This finding is consistent with other studies suggesting that the effect of EMS may be closely related to the functional engagement of the muscles and their role in the exercise.

### 4.3. Implications for Training

The significant improvements in jump smash performance and static squat jump height, coupled with increased activation in the RF, suggest that EMS combined with strength training could be an effective training strategy for improving lower limb explosiveness in athletes, particularly in sports that require rapid, powerful movements like badminton. The combination of EMS and traditional strength training allows for a more comprehensive approach to muscle activation, particularly in targeting fast-twitch muscle fibers that are critical for explosive performance [42].

For athletes in sports like badminton, where explosive movements such as jumping and rapid changes in direction are essential, the addition of EMS could offer a valuable tool for enhancing performance. EMS training can be particularly useful for athletes looking to increase their power output without overloading the muscles with heavy weights, thus reducing the risk of injury and promoting more efficient muscle recruitment [43]. Strategies combining electrical stimulation (EMS) with traditional strength training offer significant advantages in improving explosive power and athletic performance in athletes. Particularly for sports like badminton, which require fast and powerful jumps and directional changes, EMS can effectively improve explosive lower limb strength by enhancing the activation of fast muscle fibers. It has been found that EMS is able to improve muscle explosiveness and coordination through direct stimulation of motor units, especially fast-twitch muscle fibers, while avoiding the risk of overloading and injury that may be associated with the use of traditional weight training [44]. In addition, EMS training is able to reduce muscle fatigue and recovery time compared to weight training alone, thereby increasing the effectiveness and frequency of training. When performed in isolation from traditional strength training, EMS is able to improve training efficiency by optimizing muscle recruitment and reducing excessive stress on joints and ligaments. This is consistent with the results of many studies showings that the strategy of EMS combined with strength training is an efficient and safe training method to help athletes better enhance their performance in competitive sports, especially in sports that require high levels of explosiveness and quick reactions.

### 4.4. Limitations and Future Research

While the findings of this study are promising, it is important to acknowledge several limitations. First, the sample size of 15 badminton players may not be large enough to generalize the results to all athletes. Future studies with larger, more diverse participant groups are needed to confirm these findings. Additionally, while this study focused on the effects of EMS on jump performance, it would be valuable to explore its impact on other aspects of athletic performance, such as agility, endurance, and recovery. Further research could also investigate the optimal parameters for EMS (e.g., pulse frequency, intensity, and duration) to maximize performance gains.

## 5. Conclusions

In conclusion, this study provides evidence that electrical stimulation combined with strength training can significantly enhance jump performance and lower limb muscle activation in badminton players. The EMS + strength condition improved jump smash and static squat jump heights, with increased activation observed in the RF muscle. These findings support the use of EMS as a supplemental training modality for enhancing explosiveness in sports requiring high-intensity, power-driven movements. Further research is needed to explore the long-term effects and optimal use of EMS in athletic training.

## Figures and Tables

**Table 1:** Demographic information of participants.

Gender	Age (Years)	Height (cm)	Weight (kg)	Training Experience (Years)
Male	17.7 ± 1.6	186.8 ± 7.5	76.3 ± 9.6	4.5 ± 0.3

**Table 2:** Differences in badminton jump performance under different conditions.

	Baseline	Strength Only	EMS + Strength	*F*	*p*
Jump Smash	51.34 ± 3.45	52.25 ± 2.69 ^a^	53.22 ± 3.76 ^ab^	3.39	0.042
Static Squat Jump	53.76 ± 4.19	55.57 ± 3.25 ^a^	56.32 ± 2.98 ^ab^	3.67	0.033
Eccentric-Concentric Squat Jump	127.66 ± 1.23	129.38 ± 2.31	125.25 ± 1.54	0.59	0.561

Note: ^a^ represents a significant difference compared to the baseline. ^b^ represents a significant difference compared to the strength-only condition.

**Table 3:** Differences in EMG mean values during static squat jump under different conditions.

	Baseline	Strength Only	EMS + Strength	*F*	*p*
VL	0.48 ± 0.57	0.51 ± 0.76	0.60 ± 0.91	1.48	0.238
RF	0.42 ± 0.48	0.46 ± 0.38	0.63 ± 0.26 ^ab^	3.44	0.040
VM	0.46 ± 0.27	0.55 ± 0.29	0.57 ± 0.31	2.01	0.160
BF	0.49 ± 0.23	0.61 ± 0.41	0.55 ± 0.38	1.16	0.316

Note: VL: vastus lateralis, RF: rectus femoris, VM: vastus medialis, BF: biceps femoris. ^a^ represents a significant difference compared to the baseline. ^b^ represents a significant difference compared to the strength-only condition.

**Table 4:** Differences in root mean square (RMS) amplitude during static squat jump under different conditions.

	Baseline	Strength Only	EMS + Strength	*F*	*p*
VL	0.59 ± 0.32	0.61 ± 0.32	0.65 ± 0.41	1.13	0.322
RF	0.54 ± 0.36	0.56 ± 0.35	0.74 ± 0.27 ^ab^	3.66	0.033
VM	0.56 ± 0.39	0.63 ± 0.37	0.68 ± 0.22	1.90	0.141
BF	0.65 ± 0.35	0.66 ± 0.32	0.67 ± 0.35	0.74	0.482

Note: VL: vastus lateralis, RF: rectus femoris, VM: vastus medialis, BF: biceps femoris. ^a^ represents a significant difference compared to the baseline. ^b^ represents a significant difference compared to the strength-only condition.

**Table 5:** Differences in integrated EMG during static squat jump under different conditions.

	Baseline	Strength Only	EMS + Strength	*F*	*p*
VL	124.43 ± 65.63	106.38 ± 61.72	122.24 ± 75.98	1.47	0.239
RF	72.39 ± 65.73	105.93 ± 83.15	111.62 ± 70.38	2.15	0.135
VM	77.94 ± 93.05	96.57 ± 87.76	122.35 ± 85.36	1.88	0.157
BF	19.89 ± 14.05	27.25 ± 20.69	20.19 ± 9.78	0.97	0.325

Note: VL: vastus lateralis, RF: rectus femoris, VM: vastus medialis, BF: biceps femoris.

**Table 6:** Differences in EMG mean values during eccentric-concentric squat jump under different conditions.

	Baseline	Strength Only	EMS + Strength	*F*	*p*
VL	0.61 ± 0.41	0.55 ± 0.37	0.47 ± 0.31	0.05	0.952
RF	0.64 ± 0.25	0.47 ± 0.33	0.41 ± 0.25	0.17	0.840
VM	0.56 ± 0.23	0.56 ± 0.39	0.43 ± 0.37	0.07	0.936
BF	0.54 ± 0.37	0.62 ± 0.46	0.48 ± 0.22	0.47	0.495

Note: VL: vastus lateralis, RF: rectus femoris, VM: vastus medialis, BF: biceps femoris.

**Table 7:** Differences in root mean square (RMS) amplitude during eccentric-concentric squat jump under different conditions.

	Baseline	Strength Only	EMS + Strength	*F*	*p*
VL	0.63 ± 0.41	0.61 ± 0.33	0.59 ± 0.38	0.03	0.970
RF	0.72 ± 0.26	0.54 ± 0.34	0.58 ± 0.35	0.05	0.955
VM	0.61 ± 0.21	0.67 ± 0.31	0.55 ± 0.34	0.57	0.569
BF	0.62 ± 0.35	0.63 ± 0.48	0.63 ± 0.36	0.01	0.901

Note: VL: vastus lateralis, RF: rectus femoris, VM: vastus medialis, BF: biceps femoris.

**Table 8:** Differences in integrated EMG during eccentric-concentric squat jump under different conditions.

	Baseline	Strength Only	EMS + Strength	*F*	*p*
VL	114.54 ± 76.71	120.76 ± 82.86	137.13 ± 64.19	0.05	0.949
RF	105.73 ± 63.34	105.71 ± 74.21	109.15 ± 63.25	0.12	0.912
VM	77.44 ± 61.33	93.29 ± 63.91	123.65 ± 65.73	0.20	0.819
BF	23.71 ± 21.06	26.17 ± 36.87	28.91 ± 9.27	0.08	0.892

Note: VL: vastus lateralis, RF: rectus femoris, VM: vastus medialis, BF: biceps femoris.

## Data Availability

The data that support the findings of this study are available on request from the corresponding author.
